# Association between Timing of COVID-19 Diagnosis in Pregnancy and Maternal-Fetal Outcomes: A Retrospective Study

**DOI:** 10.51894/001c.140342

**Published:** 2025-05-30

**Authors:** Sathiyakala Rajendiran, Caitlin Brazda, Morgan A. Dalm

**Affiliations:** 1 Department of Obstetrics and Gynecology Gadsden Regional medical center, Gadsden, AL-35903, USA.; 2 Department of Obstetrics and Gynecology Trinity Health, Muskegon, Michigan-49444, USA.; 3 Trinity Health, Muskegon, Michigan-49444, USA

**Keywords:** pregnancy, maternal outcomes, fetal outcomes, timing of diagnosis, COVID-19

## Abstract

**OBJECTIVE:**

To examine the relationship between COVID-19 diagnosis timing during pregnancy and adverse maternal and fetal outcomes.

**METHODS:**

Pregnant women diagnosed with COVID-19 by a nasopharyngeal swab SARS-CoV-2 PCR between January 1, 2021, and December 31, 2021, irrespective of the pregnancy outcome, were included in the study. Patients not diagnosed with COVID-19 were included as a comparison group. The timing of COVID diagnosis was categorized by trimester (first trimester, <13 weeks; second trimester, 13 to <27 weeks; third trimester, >27 weeks). Maternal outcomes included placental abnormalities, HELLP syndrome, deep vein thrombosis, pulmonary embolism, and maternal death. Fetal outcomes included pregnancy loss, intrauterine growth restriction, preterm birth, stillbirth, and admission to the NICU. Quantitative data were analyzed using a one-way ANOVA and are presented as mean ± standard deviation (SD). Nominal data were compared using chi-square or Fisher’s exact tests and are reported as frequency (percent). Statistical significance was set at p < 0.05.

**RESULTS:**

A total of 289 COVID-affected pregnancies and 1706 non-COVID-19 pregnancies were included. Most patients, 189 (65.4%), were diagnosed with COVID-19 in the third trimester, 66 (22.8%) in the second trimester, and 34 (11.8%) in the first trimester. There was a statistically significant higher proportion of patients experiencing placental abnormalities in patients diagnosed with COVID-19 in the 3rd trimester with lowest occurrence in non-COVID-19 pregnancies, followed by patients diagnosed in the 1st and 2nd trimesters (p<0.001). Further, preterm delivery followed a similar frequency pattern, occurring most often in patients diagnosed with COVID-19 in the 3rd trimester (p<0.001).

**CONCLUSION:**

Patients with COVID-19 infection in the third trimester of pregnancy face a heightened risk of adverse maternal-fetal outcomes. Further investigation into this relationship is warranted.

## 1. INTRODUCTION

The global crisis caused by COVID-19 in 2020 has highlighted the susceptibility of pregnant women during emergencies and infectious diseases. Studies indicate that pregnant women who are infected with COVID-19 are more likely to face a severe form of the illness, with approximately 25% developing pneumonia and a higher risk of mortality, reaching up to 35%.^1^ Hospitalization rates for pregnant women with COVID-19 are also higher compared to non-pregnant women of the same age, especially in the later stages of pregnancy, particularly the third trimester or peripartum.^2,3^ Physiological and immunological changes during pregnancy can contribute to the increased severity of COVID-19 in pregnant women. Furthermore, there is a potential risk of the virus being transmitted from the mother to the fetus, leading to placental infection or a range of complications (e.g., restricted fetal growth, premature delivery, fetal distress, cesarean delivery, NICU admission, early-onset neonatal sepsis, or even abortion).^4–6^

Some studies have reported that the effect of COVID-19 infection through pregnancy can vary depending on the trimester in which the infection occurs. Seif et al. reported that patients infected in the second or third trimester had a higher likelihood of being admitted to the hospital and needing oxygen therapy than patients infected in the first trimester. They also reported that the rates of preterm birth (PTB) and extreme preterm birth were higher in patients infected in the first trimester. Infants born to mothers infected in the 2nd trimester had a higher percentage of neonatal sepsis workups compared to the other groups.^7^ Fallach et al. analyzed the preterm birth (PTB) rates and small-for-gestational-age (SGA) in a large and diverse cohort based on the trimester of COVID infection. They concluded that there was an elevated risk of PTB for those infected during late pregnancy, especially in patients who experienced symptoms.^8^

In contrast, other studies report no direct link between the timing of COVID-19 diagnosis during pregnancy and pregnancy outcomes. Schell et al. found that most cases of COVID-19 in pregnant women were either asymptomatic or mild.^9^ The study reported that the composite obstetrical outcome, which included preterm delivery, preeclampsia with severe features, placental abruption, excessive blood loss at delivery, and stillbirth, did not significantly differ based on the trimester of COVID-19 diagnosis. Similarly, Aydın et al. reported no significant association between the gestational age at the time of COVID-19 infection and the occurrence of adverse pregnancy outcomes such as preterm birth, pulmonary embolism, fetal growth restriction, and gestational diabetes mellitus.^10^

Due to the conflicting evidence in the literature, this study aims to examine the relationship between the timing of COVID-19 diagnosis during pregnancy (trimester of diagnosis) and its association with adverse maternal and fetal outcomes.

## 2. MATERIALS AND METHODS

A retrospective cohort study was conducted at Trinity Health Muskegon Hospital to investigate the association between the timing of COVID-19 diagnosis and maternal and fetal outcomes in pregnant women. The study received approval from the Institutional Review Board. Pregnant women diagnosed with COVID-19 by a nasopharyngeal swab SARS-CoV-2 PCR between January 1, 2021, and December 31, 2021, irrespective of the pregnancy outcome, were included in the study. Patients not diagnosed with COVID-19 were included as a comparison group. Exclusion criteria were multiple gestation, and comorbid conditions including chronic heart failure, Type I or II diabetes, chronic lung disease, immunosuppression, gestational diabetes, pre-eclampsia, gestational hypertension, and coagulation abnormalities.

Data collected included age, race, ethnicity, BMI, trimester of COVID-19 diagnosis (first trimester, <13 weeks; second trimester, 13 to <27 weeks; third trimester, >27 weeks), mode of delivery. Maternal outcomes included placenta abruption, HELLP (Haemolysis, Elevated Liver enzymes, and Low Platelets) syndrome, coagulation abnormalities, venous thromboembolism (VTE) or pulmonary embolism (PE), and maternal death. Fetal outcomes included miscarriage (pregnancy loss <20 weeks), fetal anomalies, intrauterine growth restriction (IUGR), preterm delivery (<37 weeks), NICU admission, and stillbirth (pregnancy loss ≥20 weeks).

### Statistical Analysis

Descriptive statistics were calculated. Quantitative data were analyzed using the one-way ANOVA and are expressed as the mean+SD. Nominal data were compared using chi-square or Fisher’s Exact test when appropriate and are expressed as a frequency and percent. Significance was assessed at p<0.05. Data were analyzed using Stata Statistical Software: Release 13 (College Station, TX: StataCorp LP).

## 3. RESULTS

### 3.1. Patient Characteristics

A total of 289 COVID-affected pregnancies and 1706 non-COVID-19 pregnancies were included. Most patients, 189 (65.4%), were diagnosed with COVID-19 in the third trimester, 66 (22.8%) in the second trimester, and 34 (11.8%) in the first trimester. The non-COVID-19 and COVID-19 trimester groups were similar regarding age; however, race and ethnicity were significantly different across groups (Table 1).

**Table 1.** Demographic characteristics by group

**Patient Characteristics**		**Non-COVID-19 patients (n=1706)**	**Time of COVID-19 Diagnosis**			
			**1^st^ trimester 0 to <13 weeks (n=34)**	**2^nd^ trimester 13 to <27 weeks (n=66)**	**3^rd^ trimester >27 weeks (n=189)**	**p-value**
Age (years)		27.3+5.7	27.3+4.9	27.1+5.6	28.5+5.6	0.062
Race^a^	Black	274 (16.6)	9 (30.0)	17 (27.0)	38 (21.3)	**<0.001**
	American Indian / Alaskan Native	4 (0.2)	0 (0.0)	1 (1.6)	3 (1.7)	
	Asian	0 (0.0)	1 (3.3)	2 (3.2)	9 (5.1)	
	Hawaiian / Pacific Islander	4 (0.2)	1 (3.3)	1 (1.6)	2 (1.1)	
	Multi-racial	48 (2.9)	0 (0.0)	1 (1.6)	1 (0.6)	
	White	1323 (80.0)	19 (63.3)	41 (65.1)	125 (70.2)	
Ethnicity^b^	Hispanic, Latino/a	0 (0.0)	9 (27.3)	7 (11.3)	22 (12.5)	**<0.001**
	Other Hispanic, Latino/a, Spanish origin	152 (9.3)	0 (0.0)	0 (0.0)	0 (0.0)	
	Mexican, Mexican American, Chicano/a	10 (0.6)	0 (0.0)	0 (0.0)	0 (0.0)	
	Not Hispanic, Latino/a	1477 (90.1)	24 (72.7)	55 (88.7)	154 (87.5)	

^a^unknown race frequency (%): non-COVID-19 53 (3.1); 1^st^ trimester 4 (14.3); 2^nd^ trimester 3 (4.6); 3^rd^ trimester 11 (6.2)

^b^unknown ethnicity frequency (%): non-COVID-19 67 (3.9); 1^st^ trimester 1 (3.0); 2^nd^ trimester 4 (6.1); 3^rd^ trimester 13 (6.9).

### 3.2. Maternal Adverse Outcomes

There was a statistically significant difference in the proportion of patients experiencing at least one adverse event among the groups, with the lowest proportion in the non-COVID 19 patients and the highest in the patients diagnosed with COVID-19 in the 3rd trimester (composite outcome, p<0.001; Table 2). This finding is driven by the most common adverse outcome experienced by all groups, placental abnormalities (p<0.001; Table 2).

### 3.3. Fetal Adverse Outcomes

There was no significant difference in the proportion of pregnancies with at least one adverse fetal outcome among the groups (composite outcome, Table 2). There were more frequent NICU admissions in babies born to patients diagnosed in the 2nd trimester (p<0.001; Table2). Pregnancy loss was significantly more likely to occur in patients diagnosed in the first trimester compared to 2^nd^ trimester patients diagnosed before 20 weeks and non-COVID-19 (p=0.026; Table 2). Preterm delivery occurred most often in patients diagnosed with COVID-19 in the 3^rd^ trimester, with lowest occurrence in non-COVID-19 pregnancies, followed by patients diagnosed in the 1^st^ and 2^nd^ trimesters (p<0.001; Table 2). Similar frequencies of cesarean sections were performed among the groups.

**Table 2.** Maternal and fetal outcomes

**Outcomes**	**Non-COVID-19 patients (n=1706)**	**Time of COVID-19 Diagnosis**			**p-value**
		**1^st^ trimester 0 to <13 weeks (n=34)**	**2^nd^ trimester 13 to <27 weeks (n=66)**	**3^rd^ trimester >27 weeks (n=189)**	
**Maternal Outcomes^a^**					
Composite outcome	20 (1.3)	2 (7.1)	10 (15.2)	37 (19.6)	**<0.001**
Placental abnormalities	12 (0.8)	2 (7.1)	8 (12.1)	34 (18.0)	**<0.001**
HELLP syndrome	0 (0.0)	0 (0.0)	1 (1.5)	0 (0.0)	0.051
VTE/PE	8 (0.5)	0 (0.0)	1 (1.5)	1 (0.5)	0.470
Maternal death	0 (0.0)	0 (0.0)	0 (0.0)	2 (1.1)	**0.025**
**Fetal Outcomes**					
Composite outcome^b^	184 (16.5)	2 (8.7)	14 (27.5)	28 (21.7)	0.069
NICU admissions^c^	0 (0.0)	0 (0.0)	4 (6.2)	0 (0.0)	**<0.001**
Still birth^a^	7 (0.5)	0 (0.0)	1 (1.5)	3 (1.6)	0.137
IUGR^a^	77 (5.0)	0 (0.0)	1 (1.5)	8 (4.2)	0.530
Preterm delivery (<37 weeks)^a,d^	100 (6.5)	2 (7.1)	8 (12.1)	17 (24.6)	**<0.001**
Cesarean delivery^a^	427 (27.7)	5 (17.9)	15 (22.7)	60 (31.7)	0.317
Miscarriage^e^	165 (9.7)	6 (17.6)	0 (0.0)	-	**0.026**

^a^excludes pregnancies ending in miscarriage

^b^composite excludes pregnancies ending in miscarriage and cesarean delivery

^c^excludes patients who experienced miscarriage or still birth

^d^excludes patients diagnosed with COVID-19 >37 weeks

^e^excludes patients diagnosed after 20 weeks

## 4. DISCUSSION

In this study, 65.4% of COVID-19 cases were diagnosed after 27 weeks of gestational age, which aligns with findings from previous systematic reviews and meta-analyses reporting a range of 52% - 73.9%.^11–14^ This suggests a trend of COVID-19 diagnosis occurring more frequently in the later stages of pregnancy, which may be attributed to the immunological changes that occur during later stages of pregnancy, increasing susceptibility to COVID-19 infection.

### 4.1. Effect of timing of COVID-19 infection on maternal outcomes

The current study findings revealed that experiencing at least one maternal adverse outcome was significantly more likely to occur in patients diagnosed with COVID-19 in the third trimester compared to earlier in pregnancy and non-COVID-19 patients. This finding seems to be driven by the prevalence of hypertensive disorders during pregnancy. The current study’s overall frequency of placental abnormalities in patients diagnosed with COVID-19 was 15.6%, comparable to rates reported in previous studies.^15–17^ Placental abnormalities occurred most often in patients diagnosed in the 3rd trimester. These patients were 23 times more likely to experience this adverse outcome compared to non-COVID-19 patients. A meta-analysis by Conde and Romero showed that infection with COVID-19 during pregnancy is associated with a higher risk of preeclampsia.^18^

The association between COVID-19 and an increased risk of VTE is widely recognized. However, the frequency of VTE and PE was similar among patients diagnosed with COVID and non-COVID patients in our study. Of note, the rate of VTE/PE in COVID-19 patients in our study (0.7%) aligns with a previous study conducted by Odessa and the group, as well as Badr et al (0.53%).^16,19^

Our findings revealed a maternal mortality rate in patients diagnosed with COVID-19 of 0.7%, which is similar to the rate of 0.5% reported by Mullins et al. in the PAN-COVID registry.^20^ However, other studies have reported slightly higher rates of maternal mortality.^13,21^ Notably, in this study, maternal deaths were only observed in patients diagnosed with COVID-19 in the third trimester. No maternal deaths were reported among the non-COVID-19 patients or patients diagnosed with COVID-19 in the first and second trimesters. This discrepancy in mortality rates among trimesters could be attributed to the increased severity of COVID-19 infection during the third trimester, but needs caution in interpreting as the severity of disease could not be assessed in our study.

### 4.2. Effect of timing of COVID-19 infection on fetal outcomes

Our data revealed the frequency of pregnancies ending in miscarriage was almost twice as high in patients diagnosed with COVID-19 in the 1^st^ trimester compared to non-COVID-19 patients. A cohort study investigated the impact of SARS-CoV-2 infection in the first trimester of pregnancy on fetal development and pregnancy loss. Contrary to the present report, the study found no evidence of adverse effects on early pregnancy outcomes due to SARS-CoV-2 infection.^22^

Preterm delivery was the most frequent adverse fetal outcome in all pregnancies in our study, with the highest proportion observed in third-trimester cases (24.6%). A meta-analysis by Di Mascio reported preterm birth was the most common adverse pregnancy outcome, occurring in 25% of patients.^23^ Smith et al. observed that severe COVID-19 in late pregnancy significantly increased the risk of preterm delivery compared to no COVID-19.^24^

Our entire sample had four NICU admissions and only occurred in patients diagnosed with COVID-19 in the second trimester. This could have been due to the indirect effect of COVID-19 on the placenta and its function.

### 4.3. Limitations

The study’s limitations include its retrospective design with data from a single institution. Additionally, due to the study’s retrospective nature, we were unable to control for the severity of COVID-19 or the variant in infected patients, which could have influenced the outcomes. However, despite these limitations, our results are in line with existing literature, emphasizing the importance of enhanced monitoring for pregnant individuals infected with COVID-19, particularly in the later stages of pregnancy.

## 5. CONCLUSION

Our study findings support the importance of appropriate monitoring and management of pregnant patients with COVID-19, particularly in the later stages of pregnancy, as it appears to be associated with an increased risk of adverse outcomes in pregnant patients. Our study adds valuable information to the existing literature about the effect of the timing of COVID-19 infection on pregnancy outcomes, particularly the impact in the third trimester on preterm labor and placental abnormalities.

### Author contributions

Author- Dr. Sathiyakala Rajendiran has contributed to the conception or design of the work, acquisition, analysis of data, drafting the article, final approval of the version to be published and agreement to be accountable for all aspects of the work.

Co-Author- Dr. Caitlin Brazda has contributed to the study design, analysis of data and interpretation, reviewing the draft and final approval of the version and agreeable to be accountable for all aspects of the work.

Co-Author- Dr. Morgan Dalm has contributed to data acquisition and analysis.

### Conflicts of interest

The authors have no conflicts of interest.
